# Synergistic ELCA-aspiration-DES thrombus removal strategy—embolus impact in high-risk plaque: A case report

**DOI:** 10.1097/MD.0000000000008328

**Published:** 2017-10-20

**Authors:** Yoshihiro Imai, Takehiro Yamashita, On Topaz

**Affiliations:** a Department of Cardiology, Hokkaido Ohno Memorial Hospital, Sapporo, Hokkaido, Japan; b Duke University School of Medicine, Durham, NC, USA.

**Keywords:** aspiration, embolic complication, excimer laser coronary angioplasty, red thrombus, thin-cap fibroatheroma

## Abstract

**Rationale:**

Thin-cap fibroatheroma (TCFA) and red thrombus are suggested as a high-risk of embolic complications during percutaneous coronary intervention (PCI). Intracoronary aspiration procedures occasionally result in either an insufficient thrombus removal or provide no significant effects on TCFA.

**Patient concerns:**

A 76-year-old male underwent coronary angiography for chest pain.

**Diagnoses:**

Coronary angiography revealed a tight stenosis at the right coronary artery which resulted in treatment by PCI. Optical frequency domain imaging (OFDI) delineated a red thrombus with TCFA.

**Interventions:**

To avoid embolic complications, excimer laser coronary angioplasty (ELCA) was applied with intracoronary aspiration before drug-eluting stent (DES) implantation.

**Outcomes:**

The red thrombus was vaporized by ELCA in an energy-intensity dependent manner and subsequently removed by intracoronary aspiration. The fibrous cap of TCFA was dissected with the material beneath the cap ablated by ELCA and extensively removed by intracoronary aspiration. DES implantation and postdilatation achieved an optimal result without flow compromise. This combined synergistic strategy of ELCA-aspiration-DES yielded a successful outcome.

**Lessons:**

A synergistic embolus removal strategy combining ELCA, aspiration and DES implantation is a promising option for the treatment of high-risk plaque with potential embolic complications.

## 1 Introduction

Thin-cap fibroatheroma (TCFA) and red thrombus are suggested as a high risk of embolic complications during percutaneous coronary intervention (PCI). Intracoronary aspiration procedures occasionally result in either an insufficient thrombus removal or provide no significant effects on TCFA. Herein, we describe a hypothesis-generating case example of angina pectoris where excimer laser coronary angioplasty (ELCA) and intracoronary aspiration removed both plaque and thrombus synergistically followed by drug-eluting stent (DES) implantation which achieved a successful revascularization without embolic complications.

## 2 Methods

Informed consent to involve this patient in this report was given to him.

## 3 Case

**Figure 1:**
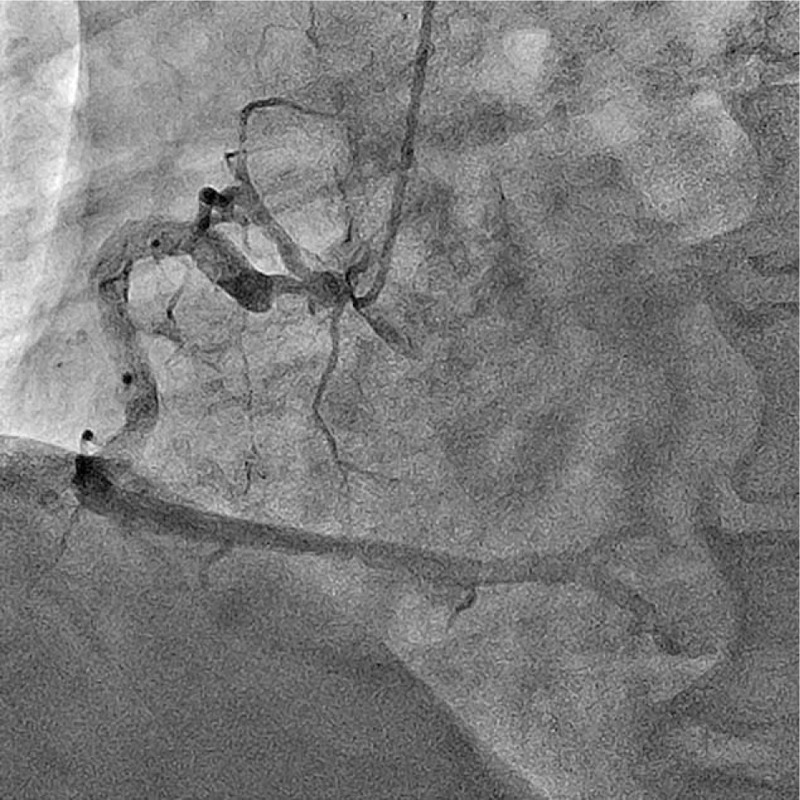
An emergency right coronary angiogram 1 year prior.

**Figure 2:**
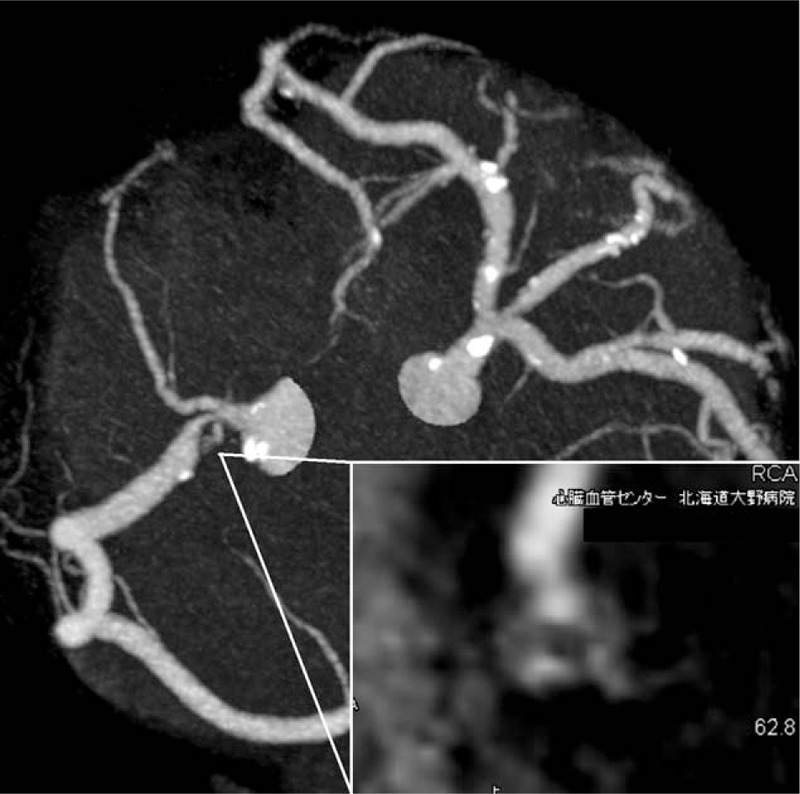
Coronary computed tomography angiography at 1-year follow-up showing an eccentric tight stenosis with a “Napkin-ring sign” at proximal RCA. RCA = right coronary artery.

**Figure 3:**
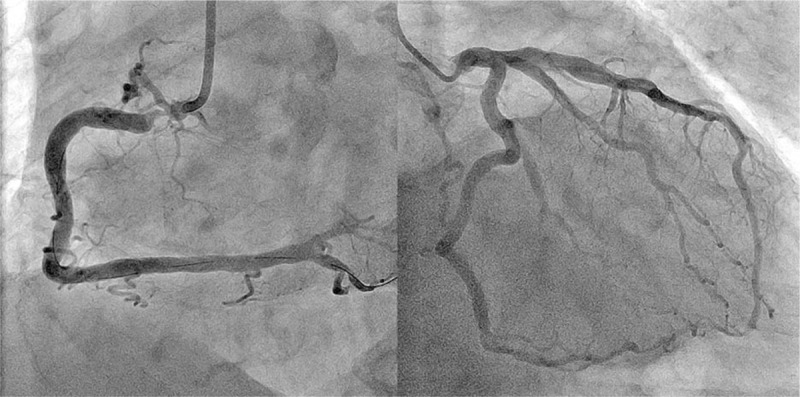
Baseline coronary angiography demonstrating a critical stenosis at the proximal RCA, with no stenosis on his left coronary system. RCA = right coronary artery.

**Figure 4:**
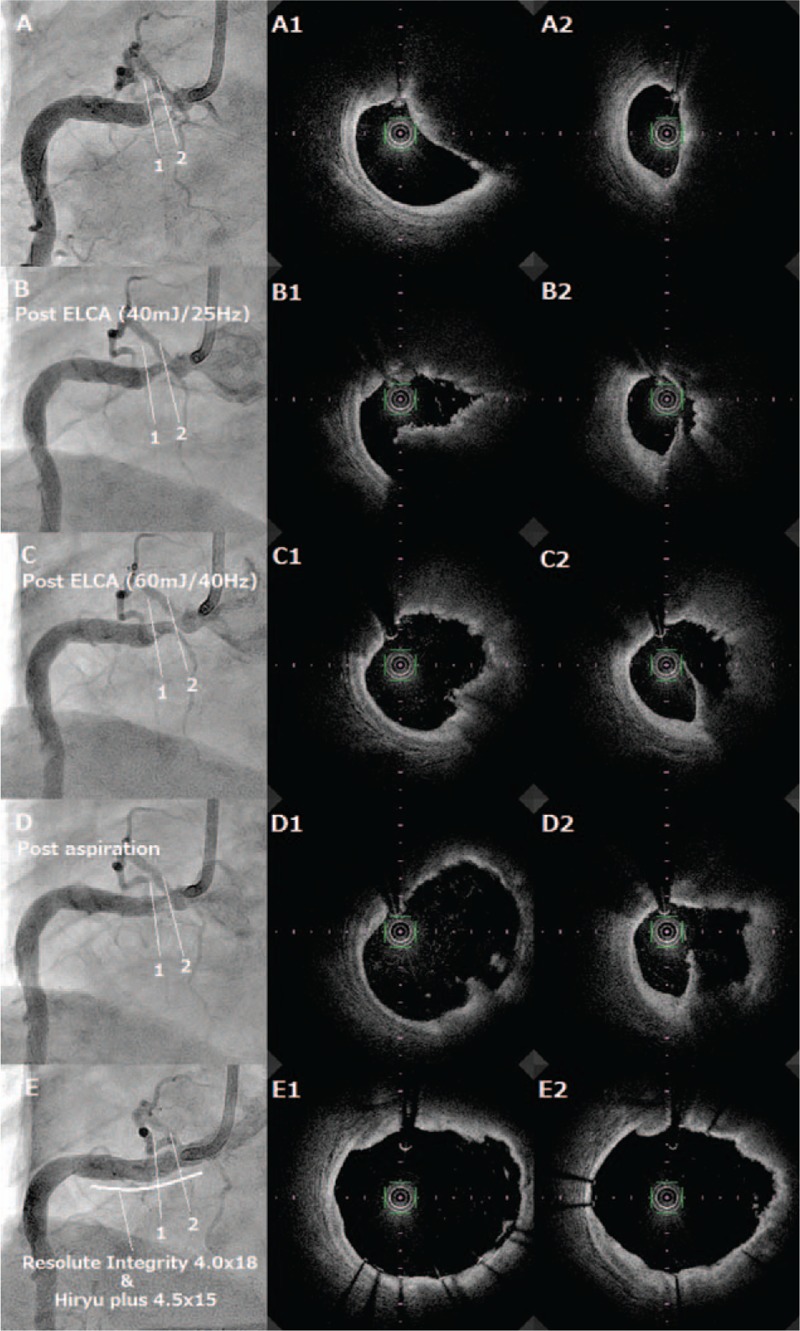
Coronary angiography (A–E) and OFDI findings (A1,2–E1,2). OFDI = optical frequency domain imaging.

**Figure 5:**
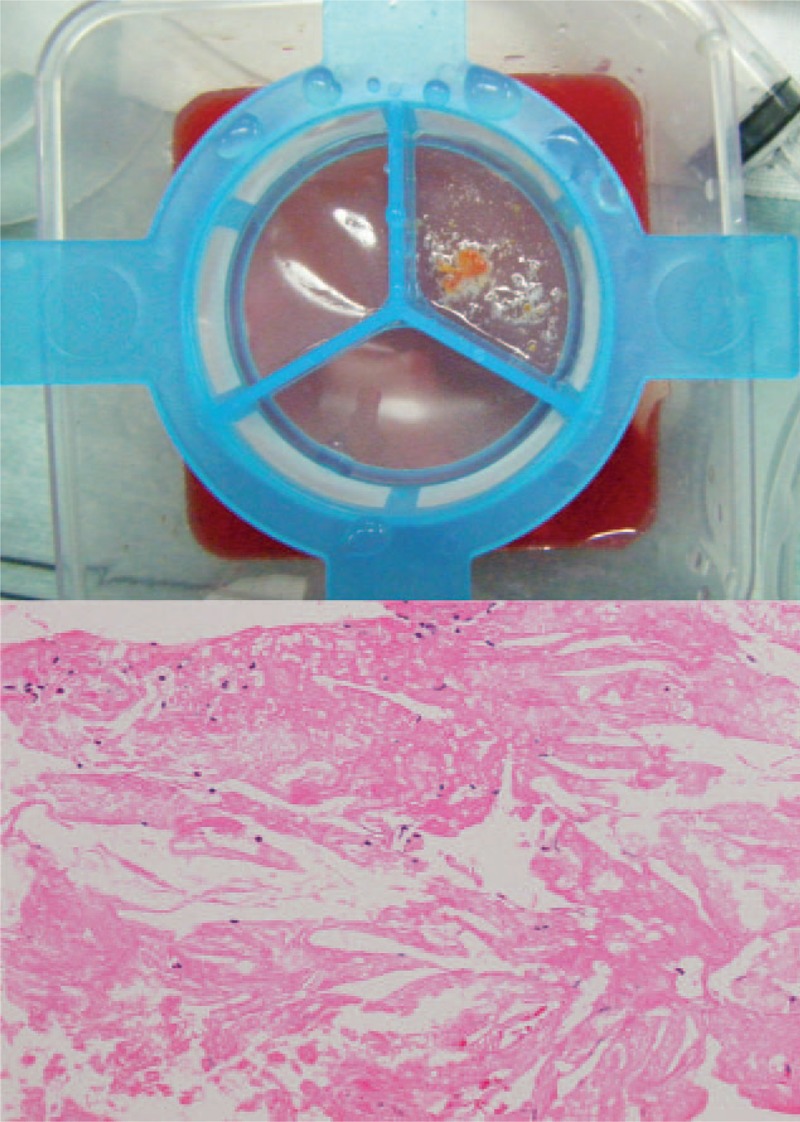
Macroscopic (upper) and microscopic (lower) findings of the aspirated material. The material appeared yellowish white. Microscopic image showed fibrin cakes with typical cholesterol clefts and foamy macrophages as a feature of atherosclerotic plaque.

A 76-year-old male had undergone an emergency coronary angiography for acute chest pain 1-year prior demonstrating a noncritical stenosis at his proximal right coronary artery (RCA) (Fig. 1), which resulted in a conservative treatment. The patient received an optimal medical therapy including aspirin and statin along with antihypertensive and antidiabetic agents. However, a follow-up coronary computed tomography angiography at 1 year demonstrated an eccentric tight stenosis with a “Napkin-ring sign” which is known as an independent risk for an occurrence of acute coronary syndrome (ACS) (Fig. 2).^[1]^ Given these findings and the fact that we were only dealing with a single vessel, it was decided to treat this lesion using PCI. During the procedure, coronary angiography demonstrated a critical stenosis at the proximal RCA, which was evaluated as tighter than previous with no stenosis on his left coronary system (Fig. 3). Optical frequency domain imaging (OFDI, FastView; TERUMO, Tokyo, Japan) delineated a TCFA proximally with red thrombus distally, both of which suggested that this lesion was a high risk for embolic complications during PCI (Fig. 4A1, A2). To avoid these risks, ELCA was applied combined with intracoronary aspiration before DES implantation. Laser atheroablation was performed using a 1.4 mm Vitesse-Cos RX catheter (Spectranetics, Colorado Springs, CO) with an incremental energy setting, starting with 40 mJ/25 Hz and completing with 60 mJ/40 Hz, followed by intracoronary aspiration using a 7F Eliminate catheter (TERUMO, Japan), which resulted in unique OFDI appearances (Fig. 4B1,2–D1,2). The red thrombus was vaporized by ELCA in an energy-intensity dependent manner and was subsequently removed by intracoronary aspiration (Fig. 4B1–D1). The fibrous cap of TCFA was dissected with the material beneath the cap ablated by ELCA and extensively removed by intracoronary aspiration (Fig. 4B2–D2). After treating the stenosis with intracoronary aspiration, D1 (Fig. 4) lumen had an area of 14.3 mm^2^ with 7.0 mm^2^ that of D2 (Fig. 4), indicating greater than 50% residual plaque in the lesion which has been shown to be an independent risk factor for restenosis.^[2]^ To reduce this risk of restenosis, implantation of a 4.0 mm × 18 mm Resolute Integrity stent (Medtronic, Minneapolis, MN) was performed, followed by postdilatation with 4.5 mm × 15 mm Hiryu plus balloon (TERUMO, Japan) at 20 ATM, resulting in an optimal angiographic result without flow compromise (Fig. 4E). The final OFDI delineated that a large and clear lumen was obtained with minimal inter-strut protruding tissues observed (Fig. 4E1 and E2). The aspirated material was macroscopically yellowish white and the microscopic findings were consistent with an atherosclerotic plaque component (Fig. 5). This combined synergistic strategy of ELCA-aspiration-DES yielded a successful outcome.

## 4 Discussion

Using computed tomography and OFDI, the lesion in this case exhibited 5 distinct features—a High-Grade Stenosis, Low Hounsfield Units, a Napkin Ring Sign, Spotty Calcium, and a TCFA. According to past studies,^[1,3,4]^ these features presented a danger of promoting ACS in the future and a high risk of distal embolization during the PCI procedure, so it was decided to treat this lesion in a timely manner using an aspiration strategy. Following careful consideration, it was resolved to maximize the effect of an embolus removal strategy by performing ELCA antecedent to an aspiration procedure. This procedural strategy was adopted because past studies^[5,6]^ have indicated that, when compared with standard balloon predilatation, routine thrombus aspiration for ST elevation myocardial infarction (STEMI) does not reduce prestent thrombus burden and fails to improve longer-term clinical outcomes while, in terms of thrombolysis in myocardial infarction (TIMI) flow grade and myocardial blush grade (MBG), another study^[7]^ demonstrated that thrombus aspiration with distal protection was superior when compared with aspiration alone. Although these studies^[5–7]^ related to STEMI, the combination of these evidences clearly indicated that even after aspiration the risk of a significant amount of residual embolic material remaining meant using the best available protection procedures to prevent such embolic complications. Attempts were made to check for any previous reports addressing the efficacy of using an aspiration procedure in patients with stable coronary artery disease but none were found. Additionally, there were no reports to support a synergistic embolus removal strategy by combining laser atheroablation and aspiration but, in this case, post procedural serial OFDI findings clearly demonstrated that the application of antecedent laser atheroablation maximized the effect of our embolus removal strategy. This strategy—using a combination of ELCA, aspiration and DES implantation—resulted in a clear and smooth lumen without any inter-strut tissue protrusion, which should also minimize the need for target lesion revascularization over the long term.^[8]^

## 5 Conclusion

A synergistic embolus removal strategy combining ELCA, Aspiration, and DES implantation is a promising option for the treatment of high-risk plaque with potential embolic complications.

## Acknowledgment

The authors also thank Mr Fred A. Lewis for his input in the proofreading of this manuscript.

## References

[1] Puchner SB Liu T Mayrhofer T High-risk plaque detected on coronary CT angiography predicts acute coronary syndromes independent of significant stenosis in acute chest pain: results from the ROMICAT-II trial. J Am Coll Cardiol 2014;64:684–92. 25125300 10.1016/j.jacc.2014.05.039 PMC4135448

[2] Honda Y Paul GY Peter JF Impact of residual plaque burden on clinical outcomes of coronary interventions. Catheter Cardiovasc Interv 1999;46:265–76. 10348121 10.1002/(SICI)1522-726X(199903)46:3<265::AID-CCD3>3.0.CO;2-8

[3] Nishio M Ueda Y Matsuo K Association of target lesion characteristics evaluated by coronary computed tomography angiography and plaque debris distal embolization during percutaneous coronary intervention. Circ J 2014;78:2203–8. 24998191 10.1253/circj.cj-14-0103

[4] Lee T Yonetsu T Koura K Impact of coronary plaque morphology assessed by optical coherence tomography on cardiac troponin elevation in patients with elective stent implantation. Circ Cardiovasc Interv 2011;4:378–86. 21791670 10.1161/CIRCINTERVENTIONS.111.962506

[5] Bhindi R Kajander OA Jolly SS Culprit lesion thrombus burden after manual thrombectomy or percutaneous coronary intervention-alone in ST-segment elevation myocardial infarction: the optical coherence tomography sub-study of the TOTAL (ThrOmbecTomy versus PCI ALone) trial. Eur Heart J 2015;36:1892–900. 25994742 10.1093/eurheartj/ehv176 PMC5061563

[6] Jolly SS Cairns JA Yusuf S Outcomes after thrombus aspiration for ST elevation myocardial infarction: 1-year follow-up of the prospective randomised TOTAL trial. Lancet 2016;387:127–35. 26474811 10.1016/S0140-6736(15)00448-1 PMC5007127

[7] Satoh S Inoue H Omura S Comparison of the reperfusion efficacy of thrombus aspiration with and without distal protection during primary percutaneous coronary intervention in patients with acute ST-segment elevation myocardial infarction. Am J Cardiol 2013;112:1725–9. 24035161 10.1016/j.amjcard.2013.07.039

[8] Soeda T Uemura S Park SJ Incidence and clinical significance of poststent optical coherence tomography findings: one-year follow-up study from a multicenter registry. Circulation 2015;132:1020–9. 26162917 10.1161/CIRCULATIONAHA.114.014704

